# Supplementary material for: Association of TP53 with Defective Long Chain 3-Hydroxy acyl-CoA Dehydrogenase Induced Non-Cirrhotic Hepatocellular Carcinoma
Source: Cancers (Basel). 2025 Oct 6;17(19):3241. doi: 10.3390/cancers17193241 (PMC12524079; doi:10.3390/cancers17193241)
Supplement: Supplementary file 1 [file cancers-17-03241-s001.zip › cancers-3808532-supplementary.pdf]

Table S1. LTQ-Orbitrap LC-MS/MS data of liver proteins that were expressed significantly different (< 0.01)) in heterozygous LCHAD mouse as compared to Wild-type mouse. Spot numbers are as assigned in Figure 4B. Up-regulated proteins are shown with HT/WT ratio of more than 1 and down-regulated proteins with HT/WT ratio of less than 1. HT represents protein visible in only heterozygous mouse liver 2D gel image.

Spot	Protein ids	Accession #	% Sequence coverage	# of unique peptides	Mr (Da) Theoretical	Ratio HT/WT	Total Ion score(sequest X corr score)	Location in the cell	*p*-Value
159	Glyceraldehyde-3-phosphate dehydrogenase-like isoform 2 (GAPDH)	gi|149273202	40	13	35,810	1.81	712	cytoplasm	0.001
132	Heat shock protein 9A or Stress- 70 protein (HSPA9)	gi|148664706	56	42	73,462	1.71	1778	Mitochondria	0.01
128	GMP synthase (glutamine-hydrolyzing) (GMPS)	gi|145558920	50	31	76,725	HT	191	Cytoplasm	0.001
129	TNF receptor associated protein1 (TRAP1)	gi|148664808	61	47	81,208	HT	1513	Mitochondria	0.002
135	Cysteine-sulfinic acid decarboxylase, isoform X5 (CSAD)	gi|568992511	50	27	55,145	HT	803	Cytoplasm	0.01
141	Protein NDRG2, isoform X2 (NDRG2)	gi|568987856	37	11	45,186	0.228	127	Nucleus	0.001
140	Arsenite methyl transferase (51%) (AS3MT)	gi|119392105	51	20	41,793	0.278	239	Nucleus & Nucleoli	0.01
136	Proliferation-associated protein 2G4 (PA2G4)	gi|148692638	56	22	43,698	0.302	411	Cytoplasm & Nucleus	0.001
127	Methylcrotonoyl-CoA carboxylase subunit alpha (MCC1)	gi|186700620	63	37	79,345	0.343	573	Mitochondria	0.01
138	Methionine adenosyltransferase I, alpha (MAT1A)	gi|148692971	30	10	43,508	0.351	162	Intracellular	0.001
145	Indoleamine-2,3-dioxygenase like protein (IDO2)	gi|132424406	59	19	45,256	0.352	275	Cytoplasm	0.001
146	Long chain specific acyl-CoA dehydrogenase (ACADL)	gi|32130423	39	17	47,908 or 47,891	0.387	251	Mitochondria	0.01
187	Copper chaperone for superoxide dismutase (CCS)	gi|20072487	25	5	28,893	0.445	92	Mitochondria	0.01

156	Nitrilase 1, isoform CRA_a (NIT1)	gi|148707145	62	16	31,886	0.456	366	Cytoplasm & nucleus	0.001
133	Dihydrolipoamide acetyltransferase component of PDH complex (DLAT)	gi|146325018	36	19	67,942	0.461	266	Mitochondria	0.002
151	Malate dehydrogenase 1 NAD (soluble), isoform CRA_c (MDH1)	gi|148675904	43	18	40,060	0.478	398	Cytoplasm	0.001
186	Cytochrome C1, heme protein (CYC1)	gi|13385006	36	9	35,328	0.51	162	Mitocondria	0.01
178	Isoform 2 or b of alpha-aminoadipic semialdehyde dehydrogenase (ALDH7A1)	gi|188035915	36	17	55,645	0.513	188	Mitochondria & Cytosol	0.01
188	Tyrosine 3-monooxygenas/tryptophan 5-monooxygenase activation protein/ epsilon polypeptide (YWHAE)	gi|148680892	64	17	29,175	0.537	497	???	0.01
176	Annexin A6 isoform A (ANXA6)	gi|31981302	57	34	75,889	0.553	527	Intracellular	0.01
155	L-Aspartate dehydrogenase, mcG8752, isoform CRAb (ASPDH)	gi|73622214	65	16	30,251	0.556	532	Nucleus	0.001
139	Succinyl-CoA ligase[ADP-forming] subunit beta, isoform CRA_b (SUCLG2)	gi|148703906	67	32	47,901	0.561	1303	Mitochondria	0.01
153	Catechol-O-methyltransferase 1 (COMT)	gi|14714535	65	15	29,487	0.571	221	Cytoplasm & Golgi Apparatus	0.002
192	Cytochrome c oxidase subunit 5A (COX5A)	gi|148693959	27	5	16,101	0.747	246	Mitochondria	0.01
